# The effects of self-care education based on the health literacy index on self-care and quality of life among menopausal women: a randomized clinical trial

**DOI:** 10.1186/s12905-022-02007-2

**Published:** 2022-11-16

**Authors:** Zahra Hossein Mirzaee Beni, Raziyeh Maasoumi, Shahzad Pashaeypoor, Shima Haghani

**Affiliations:** 1 Department of Community Health Nursing, School of Nursing and Midwifery, Tehran University of Medical Sciences, Tehran, Iran; 2 Department of Reproductive Health, School of Nursing and Midwifery, Tehran University of Medical Sciences, Tehran, Iran; 3 Nursing and Midwifery Care Research Center, School of Nursing and Midwifery, Tehran University of Medical Sciences, Tehran, Iran; 4 Deptartment of Community Health and Geriatric Nursing, School of Nursing and Midwifery, Tehran University of Medical Sciences, Tehran, Iran; 5 Community Based Participatory Research Center, Iranian Institute for Reduction of High - Risk Behaviors, Tehran University of Medical Sciences, Tehran, Iran; 6 Nursing Care Research Center, Iran University of Medical Sciences, Tehran, Iran

**Keywords:** Self-care, Quality of life, Menopause

## Abstract

**Purpose:**

Aging is associated with many different health-related challenges for women such as menopause and its associated problems. Self-care (SC) is a factor with potential effects on menopause and its consequences. SC education based on health literacy has the potential to improve menopausal women’s SC. The aim of this study was to evaluate the effects of SC education based on the health literacy index (HLI) on SC and quality of life (QOL) among menopausal women.

**Methods:**

This randomized clinical trial was conducted in Iran. Participants were 100 menopausal women purposively recruited from five comprehensive healthcare centers in the south of Tehran. They were randomly allocated to a control and an intervention group through block randomization. Intervention was an HLI-based SC education program implemented in four 1.5–hour weekly sessions through the lecture, group discussion, and question and answer methods. Data were collected before and eight weeks after the intervention using a demographic questionnaire, the Health Literacy for Iranian Adults scale, the Menopause-Specific Quality of Life Questionnaire, and the Menopausal Self-Care Questionnaire. The SPSS software (v. 22.0) was used to analyze the data through the Chi-square, Fisher’s exact, paired-sample *t*, and the independent-sample *t* tests as well as the analysis of covariance at a significance level of less than 0.05.

**Findings:**

There were no significant differences between the intervention and the control groups respecting the pretest mean scores of QOL (88.15 ± 32.36 vs. 79.6 ± 36.99) and SC (104.75 ± 12.31 vs. 103.32 ± 13.8) (*P* > 0.05). However, the posttest mean scores of QOL and SC in the intervention group significantly differed from the control group (66.44 ± 28.41vs. 81.3 ± 38.04 and 125.6 ± 11.23 vs. 102.6 ± 14.34) (*P* < 0.05).

**Conclusion:**

HLI-based SC education is effective in significantly improving menopausal women’s QOL and SC and can be used to improve health-related outcomes among menopausal women.

*Clinical trial registration* This research was registered (24/03/2020) in the www.thaiclinicaltrials.org with registration number: TCTR20200324002.

## Background

Menopause is a normal inevitable process in women’s life which is defined as the non-pathological absence of monthly menstruation for twelve consecutive months due to the termination of ovarian follicular activity [1, 2]. Population aging and improved life expectancy have resulted in the increasing number of menopausal women so that most women currently spend one third of their life after menopause [3, 4]. The World Health Organization estimates that there will be 1.3 billion menopausal women in the world in 2030 [4]. Estimates also show that five million menopausal women live in Iran in 2021 [5].

Normal changes in ovarian function during menopause are associated with different physical, mental, and social symptoms [6, 7]. Examples of these symptoms are hot flashes, night sweats, mood and sleep disorders, memory impairment, concentration disturbances, nervousness, depression [7, 8], dizziness, tachycardia, vaginal atrophy, bladder irritability, headache, anger [9], sexual dysfunction [7], fatigue [10], breast and skin atrophy [9], weight gain, and increased abdominal and subcutaneous fat [11]. Moreover, menopause triggers or aggravates chronic illnesses such as diabetes mellitus, osteoporosis, cardiovascular disease, and respiratory and musculoskeletal disorders which are complicated by changes in social status, children leaving home, birth of grandchildren, and death of parents [12]. All these problems affect physiological and psychological quality of life (OQL) [12, 13]. Women’s limited knowledge about menopause and their encounter with conflicting information also cause them worries about menopause-associated physical and mental changes, give them negative attitude towards menopause, and reduce their QOL [14].

Self-care (SC) is a factor with potential positive effects on menopause and its consequences and is considered as the most principal factor behind menopausal women’s health. By definition, SC is the ability of individuals, families, or communities to maintain and promote health, prevent illnesses, and cope with illnesses or disabilities with or without receiving healthcare providers’ support [15]. It is in fact a strategy to cope with life events and stressors. Advances in medical technology and increases in healthcare costs, importance of health maintenance by individuals and communities based on human needs, increasing personal responsibility towards personal health, and reduced availability of healthcare services due to population growth have turned SC into an important determinant of health and quality of life [16].

Despite the importance of SC to menopausal women’s health and QOL, studies on menopausal women reported that they had limited knowledge about menopause and SC and limited access to menopause-related information [13, 14]. For example, a study in Iran found that 45% of menopausal women had limited knowledge about SC and only 0.8% of them performed appropriate SC activities [16].

The results of some studies also show that the existing interventions have not been able to affect all aspects of self care of menopausal women. The results of studies aimed at improving the health of menopausal women through self care principles training [17] and group counseling [18] show the same claim.

Therefore, innovative SC-based educational interventions can be used to improve menopausal women’s SC ability, health status, QOL, self-efficacy, and coping with menopausal problems [16]. Adequate knowledge about menopausal problems helps reduce menopausal problems and increase QOL [18].

Health literacy (HL) is a major factor affecting SC [19]. By definition, HL is the ability to acquire, understand, and process health-related information to make appropriate health-related decisions [20]. Therefore, it is considered as an essential tool for health promotion and care quality improvement [21]. Nonetheless, previous studies reported that most people have limited HL. A study in Iran also showed that around half of the Iranians had limited HL [22].

Limited HL among healthcare clients reduces the effectiveness of educational interventions and highlights the necessity of designing educational interventions based on HL. Studies showed that HL-based education reduces the negative effects of low HL on the outcomes of educational interventions [23, 24]. In 2011, the Centers for Disease Control and Prevention developed the Health Literacy Index (HLI) to help improve the quality of educational materials for healthcare clients. HLI is a comprehensive index with 63 items in ten main criteria, namely plain language, clear purpose, graphic characteristics, user involvement, skill-based learning, audience appropriateness, instructions, development details, evaluation methods, and strength of evidence [25]. HLI is a significant predictor of health and use of healthcare services. It can be used to assess healthcare clients’ HL and their understanding of health-related information and develop appropriate and accessible need-based educational materials [23, 26].

There are limited data about the effects of HLI-based educational interventions. One of the handful studies in this area reported that HLI-based education significantly improved elderly people’s understanding of fall prevention strategies and recommended further studies to assess the effects of HLI-based education [23]. Another study showed that HLI-based patient education had significant positive effects on medical adherence among elderly people, reported the paucity of evidence regarding the effects of HLI-based education, and highlighted the necessity of further studies in this area [24]. Moreover, our literature search revealed no study into the effects of HLI-based education on menopausal women. Therefore, the present study was conducted to narrow this gap. The aim of the study was to evaluate the effects of HLI-based SC education on SC and QOL among menopausal women.

## Methods

### Design

This randomized clinical trial was conducted in Iran.

### Participants and setting

Study population consisted of all menopausal women who referred to the comprehensive healthcare centers in the south of Tehran, Iran. Eligible menopausal women were purposively recruited to the study from five centers using the name lists available in the Integrated National Health System of Iran. Inclusion criteria were a menopause history of one to five years, married life, self-report active sexual life, basic literacy skill, an HL score of less than 50 for the Health Literacy for Iranian Adults scale, no history of serious stressful events in the past month, no self-report history of mental problems, no history of hormone therapy in the last three months, and no affliction by debilitating chronic illnesses. Exclusion criteria were two consecutive absences from the intervention sessions.

**Fig. 1:**
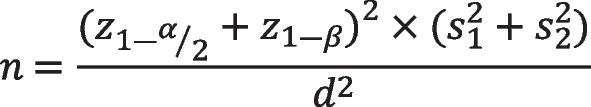
Sample size calculation formula

Sample size was calculated with a *d* of 10 [23], a power of 0.80, a confidence level of 0.95, and a probable attrition rate of 10%. The sample size calculation formula (Fig. 1) showed that fifty participants per group were necessary. Sample size calculation was performed using the standard deviations of the different aspects of QOL in a previous study [1]which revealed that the use of the standard deviation of the physical aspect of QOL was associated with the greatest sample size.

$$n=\frac{({{z}_{1-\alpha/2}+{z}_{1-\beta })}^{2}\times ({s}_{1}^{2}+{s}_{2}^{2})}{{d}^{2}}$$

Participants were allocated to a control and an intervention group through block randomization with the six blocks of AABB, ABAB, ABBA, BBAA, BABA, and BAAB. The random sequence of the blocks was determined using the randomization.com website. For allocation concealment, 25 cards labeled with the block names were prepared, each was put in an opaque bag, one bag was opened for each four participants, and they were allocated to the groups based on the allocation sequence on the card.

### Outcomes

The primary outcome of the study was QOL and the secondary outcome was SC. Data were collected using self-report instruments at two time points, namely before and eight weeks after the intervention.

### Instruments

Data collection instruments were a demographic questionnaire, the Health Literacy for Iranian Adults scale, the Menopause-Specific Quality of Life Questionnaire, and the Menopausal Self-Care Questionnaire.

The items of the demographic questionnaire were on age, menopause age, menopause duration, educational level, occupation, husband’s age and occupation, number of children, and family income.

The Health Literacy for Iranian Adults scale was developed and psychometrically evaluated by Montazeri et al. in 2015. This scale has 33 items in the five subscales of access (six items), reading skill (four items), understanding (seven items), appraisal (four items), and decision and application (twelve items). Items are scored on a five-point Likert scale as follows: 5: “Completely easy”; 4: “Easy”; 3: “Neither easy nor difficult”; 2: “Difficult”; and 1: “Very difficult”. The possible total scores of the access, reading skill, understanding, appraisal, and decision and application subscales are 30, 20, 35, 20, and 60, respectively. Subscale scores are changed to 0–100 scale and are interpreted as follows: scores 0–50: poor HL; scores 50.1–66: inadequate HL; scores 66.1–84: adequate HL; and scores 84.1–100: excellent HL. This scale has acceptable validity and reliability and a Cronbach’s alpha of 0.884 [22]. In the present study, this scale was used for eligibility assessment and women with a score of less than 50 were included.

The Menopause-Specific Quality of Life Questionnaire was developed and standardized in 1996 by Hilditch et al. in Toronto University, Canada. This questionnaire has 29 items in four main subscales, namely vasomotor (items 1–3), psychosocial (items 4–10), physical (items 11–26), and sexual (items 27–29) QOL. Scoring is performed on an eight-point scale from zero (“No experience of symptoms during the last month”) to 7 (“Experience of severe symptoms”). The possible total score of the scale is 0–203, with higher scores showing lower QOL [27]. Hilditch et al. confirmed the validity of this questionnaire using a panel of experts and its reliability with a test-retest correlation coefficient of 0.95 [27]. A study in Iran also confirmed the acceptable reliability of the questionnaire with a Cronbach’s alpha of 0.92 for the vasomotor subscale, 0.88 for the psychosocial subscale, 0.93 for the physical subscale, and 0.87 for the sexual subscale [28].

The Menopausal Self-Care Questionnaire was developed in 2017 by Kafaei-Atrian et al. and has 34 items in seven main subscales of general health (nine items), screening (six items), nutrition (six items), memory (three items), hot flashes and night sweats (three items), sexuality (three items), and social communication (four items). Items are scored on a five-point Likert scale as follows: 1: “Never”; 2: “Rarely”; 3: “Sometimes”; 4: “Often”; and 5: “Always”. The possible total scores of the general health, screening, nutrition, memory, hot flashes and night sweats, sexuality, and social communication subscales are respectively 45, 30, 25, 15, 15, 15, and 20 and the possible total score of the questionnaire is 7–165, with higher scores indicating greater SC ability. Kafaei-Atrian et al. assessed the psychometric properties of this questionnaire and found that its item impact score, content validity ratio, content validity index, test-retest intraclass correlation coefficient, and Cronbach’s alpha were 2.84, 0.70, 0.70, 0.76, and 0.88, respectively [29].

### Intervention

Study intervention was an HLI-based SC education program developed using HLI-based sources approved by the Centers for Disease Control and Prevention. Six experts in women’s health approved the validity of the program and its congruence with the ten criteria of HLI, namely plain language, clear purpose, graphic characteristics, user involvement, skill-based learning, audience appropriateness, instructions, development details, evaluation methods, and strength of evidence. Experts scored each criterion zero or one and then, the total score of the experts for the criteria were calculated in the 0–100 scale which were 100, 100, 100, 100, 93.75, and 87.5, with a total mean of 96.87. A study reported that a score of more than 60 shows the congruence of educational materials with HLI and the appropriateness of educational materials [23]. Participants in the intervention group were divided into 8–10-person groups and received education about, physical, vasomotor, psychosocial, and sexual SC in four 1.5–hour weekly sessions through the lecture, group discussion, and question and answer methods. Moreover, participants could call the study authors to ask their SC-related questions during the eight-week period between the intervention end and the posttest. Participants in the control group only received care services routinely provided to all menopausal women who referred to all comprehensive healthcare centers in Tehran, Iran.

Routine care for menopausal women in comprehensive healthcare centers included holding educational classes in the form of lecture, consultation with center’s midwife, and referral to nutrition and psychology section if necessary.

### Ethical considerations

The Ethics Committee of Tehran University of Medical Sciences, Tehran, Iran (This committee examines all studies related to research centers and faculties), approved this study (code: IR.TUMS.FNM.REC.1398.093) and the study was registered in the Thailand Registry of Clinical Trials (code: TCTR20200324002). We provided participants with information about the study aim, voluntariness of participation, and confidentiality of their data and ensured them that their refusal of participation in the study would have no effects on the receiving the available care services. An SC-related educational booklet was also provided to participants in the control group after the posttest.

### Statistical data analysis

The SPSS software (v. 22.0) was used for data analysis. Data were described using the measures of descriptive statistics and were analyzed through the Chi-square, Fisher’s exact, paired-sample *t*, and the independent-sample *t* tests as well as the analysis of covariance. The level of significance was set at less than 0.05.

## Results

**Fig. 2:**
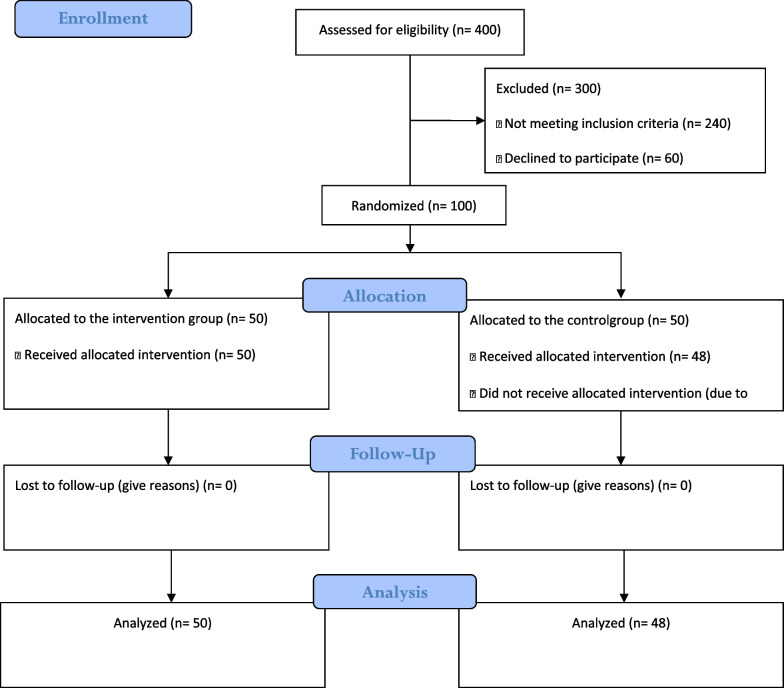
The flow diagram of the study

One hundred menopausal women were recruited to the study. Two participants from the control group were excluded due to voluntary withdrawal from the study and the study was finished with 48 participants in the control group and fifty participants in the intervention group (Fig. 2).

The means of participants’ age, menopause age, and menopause duration were respectively 52.91 ± 2.48, 50.14 ± 2.41, and 2.8 ± 1.49 years in the intervention group and 53.96 ± 2.98, 50.64 ± 2.89, and 3.36 ± 1.48 years in the control group. Most participants in the intervention and the control groups were housewife (85.4% vs. 78%). The means of health literacy (HL) were 46.8 ± 2.12 and 47.2 ± 3.17 in the intervention and control group (HL < 50 for inclusion criteria).

There were no significant differences between the groups in terms of participants’ age, menopause age, menopause duration, number of children, educational level, occupation, family income, and their husband’s age and occupation (P > 0.05; Table 1).

**Table 1:** Between-group comparisons with respect to participants’ characteristics

Characteristics	Group		
	Intervention	Control	*P* value
	N (%) or Mean±SD	N (%) or Mean±SD	
Educational level			
Primary	16 (33.3)	16 (32)	0.887*
Guidance school	13 (27.1)	12 (24)	
High school	3 (6.3)	2 (4)	
Diploma and higher	16 (33.3)	20 (40)	
Occupation			
Housewife	41 (85.4)	39 (78)	0.207*
Employed	4 (8.3)	10 (20)	
Retired	3 (6.3)	1 (2)	
Husband’s occupation			
Unemployed	2 (4.3)	5 (10)	0.24*
Employed	28 (60.9)	22 (44)	
Retired	16 (34.8)	23 (46)	
Family income			
Sufficient	3 (6.5)	6 (12.5)	0.118*
Relatively sufficient	29 (63)	20 (41.7)	
Insufficient	14 (30.4)	22 (45.8)	
Age (Years)	52.91 ± 2.48	53.96 ± 2.98	0.063^
Menopause age (Years)	2.41 ± 50.14	2.89 ± 50.64	0.352^
Menopause duration (Years)	1.49 ± 2.8	1.48 ± 3.36	0.066^
Husband’s age (Years)	4.4 ± 57.88	5.94 ± 59.68	0.09^
Number of children	1.03 ± 2.73	1.36 ± 2.76	0.9^
Health Literacy	46.8 ± 2.12	47.2 ± 3.17	0.21^

*The results of the Chi-square or the Fisher’s exact test; ^The results of the independent-sample *t* test

There was a significant difference between the groups in terms of the pretest mean score of the vasomotor subscale of QOL (*P* = 0.04), while the between-group differences respecting the pretest mean scores of QOL and its physical, psychosocial, and sexual subscales were not statistically significant (*P* > 0.05; Table 2). However, the posttest mean scores of QOL and all its subscales in the intervention group were significantly greater than the control group (*P* < 0.05; Table 2). Moreover, there were no significant differences between the study groups with respect to the pretest mean scores of SC and its subscales (P > 0.05), except for the pretest mean score of the nutrition subscale (*P* = 0.007). However, the posttest mean scores of SC and all its subscales in the intervention group were significantly higher than the control group (*P* < 0.00) (Table 3).

**Table 2:** Between- and within-group comparisons with respect to the mean scores of QOL and its subscales

QOL	Groups		
	Intervention	Control	*P* value
	Mean ± SD	Mean ± SD	
Vasomotor			
Before	8.31 ± 5.58	5.36 ± 4.34	0.004*
After	3.75 ± 4.63	5.32 ± 4.48	<0.001**
*P* value^	<0.001	0.875	–
Psychosocial			
Before	9.74 ± 20.6	11.27 ± 17.98	0.221*
After	8.47 ± 15.38	11.48 ± 18.5	<0.001**
*P* value^	<0.001	0.048	
Physical			
Before	21.01 ± 47.38	23.08 ± 44.66	0.545*
After	17.47 ± 36.17	24.01 ± 45.62	<0.001**
*P* value^	<0.001	0.19	
Sexual			
Before	11.81 ± 5.45	11.42 ± 6.831	0.754*
After	5.52 ± 11.19	11.88 ± 6.829	<0.001**
*P* value^	<0.001	0.042	
Total			
Before	32.36 ± 88.15	79.6 ± 36.99	0.227*
After	28.41 ± 66.44	81.3 ± 38.04	<0.001**
*P* value^	<0.001	0.01	

^The results of the paired-sample *t* test; *The results of the independent-sample *t* test; **The results of the analysis of covariance

**Table 3:** Between- and within-group comparisons with respect to the mean scores of SC and its subscales

SC	Groups		
	Intervention	Control	*P* value
	Mean ± SD	Mean ± SD	
General health			
Before	26.94 ± 4.99	27.12 ± 5.57	0.865*
After	36.44 ± 4.24	25.98 ± 5.85	<0.001**
*P* value^	<0.001	0.002	–
Screening			
Before	16.92 ± 5.50	4.99 ± 15.72	
After	22.06 ± 8.47	15.84 ± 3.002	<0.001**
*P* value^	<0.001	0.276	
Nutrition			
Before	18.96 ± 2.379	17.48 ± 2.91	0.007*
After	20.94 ± 2.254	17.26 ± 3.002	<0.001**
*P* value^	<0.001	0.04	
Memory			
Before	8.98 ± 2.46	9.5 ± 2.49	0.301*
After	2.33 ± 10.5	9.3 ± 2.56	<0.001**
*P* value^	<0.001	0.017	
Hot flashes and night sweats			
Before	2.91 ± 9.13	9.38 ± 3.25	0.684*
After	3.11 ± 9.5	10.2 ± 23.39	<0.001**
*P* value^	<0.001	0.322	
Sexuality			
Before	6.67 ± 2.76	6.58 ± 2.93	0.881*
After	7.21 ± 2.82	6.62 ± 2.94	<0.001**
*P* value^	<0.001	0.622	
Social communication			
Before	17.17 ± 1.64	17.54 ± 1.90	0.303*
After	23.18 ± 1.242	17.52 ± 1.83	<0.001**
*P* value^	<0.001	0.709	
Total			
Before	104.75 ± 12.31	103.32 ± 13.87	0.591*
After	125.6 ± 11.23	102.6 ± 14.34	<0.001**
*P* value^	<0.001	0.01	

^The results of the paired-sample *t* test; *The results of the independent-sample *t* test; **The results of the analysis of covariance

## Discussion

This study was conducted to evaluate the effects HLI-based SC education on SC and QOL among menopausal women. Findings revealed that the study intervention significantly improved the mean scores of QOL and SC and all its subscales.

The findings of this study indicated significant improvement in QOL and all its subscales after the study intervention. In line with this finding, a former studies reported the significant positive effects of a structured educational program based on group support on QOL among menopausal women [30]. The findings of a study that investigated the effect of the educational intervention show that the educational intervention significantly improved the quality of life of menopausal women [31]. Another study on sixty menopausal women in Iran also showed the effectiveness of an educational intervention based on HL strategies in significantly improving QOL [32]. Moreover, a study showed that HL had significant relationship with QOL among menopausal women [33].Also, in a descriptive-analytical study that predicted quality of life based on health literacy, the results showed that health literacy is an effective factor in the quality of life of postmenopausal women [34].

On the other hand, the results of studies that used other methods such as Health-Promoting Lifestyle Modification Education [35] and self-care training [36] could not be effective in all dimensions of quality of life.

The interventions of the two mentioned studies were not tailored based on the level of health literacy of the people. It seems that the lack of appropriate education with the level of health literacy and learning abilities of the research samples was a factor in not achieving the desired results in this study. The favorable results received from the current study confirmed that using the health literacy index and providing education based on the level of health literacy and learning abilities of people can improve their quality of life.

We also found that HLI-based SC education had significant positive effects on SC among menopausal women. In a study researches conducted a study to develop a health literacy index for creating accessible and readable health information material for people of all literacy levels. The results showed that using the educational content by older adults and their feedback during the validation process helped develop an improved health literacy based manual for prevention of falls [23]. In another study, self-management education based on health literacy promotes medication adherence among older adults [24].

In agreement with this finding, a previous study showed that SC education based on Orem’s Self-Care model significantly improved menopausal women’s SC [37]. Another study found the positive effects of health education on healthcare knowledge and ability of menopausal women [32]. Moreover, a study found that education based on HL strategies significantly improved SC among older adults [38]. Also, the results of a review study showed that self care training interventions can be one of the effective strategies to improve the self care ability of menopausal women [39].

On the other hand, the results of some studies showed that the educational intervention based on the health literacy index did not affect some aspects of the self care of elderly [40], which could be due to individual, cultural and religious differences, ways of thinking and different expectations from life.

Among the strengths of this study, it can be mentioned that this study is one of the few studies whose content is designed using the health literacy index and tailored to people’s health literacy. Considering the high prevalence of limited health literacy among menopausal women, tailoring educational interventions to their health literacy level can increase the effectiveness of these interventions. It is suggested to investigate the effect of using the health literacy index in the preparation and evaluation of educational contents in other areas of women’s health, other ages, and also compare the educational intervention based on the health literacy index should be done with other educational methods and in other environments.

We faced some limitations in this study such as the lack of quality educational facilities and equipment as well as limited knowledge of the authorities and staff of the study setting about the study intervention and their limited collaboration.

## Conclusion

The results of this study showed that self care education based on the health literacy index decreased the quality of life scores and increased the self-care scores of menopausal women, which means improving their quality of life and self care.

This study concludes the effectiveness of HLI-based SC education in significantly improving QOL and SC among menopausal women. Therefore, HLI can be used to evaluate the appropriateness of educational materials and improve the quality of educational programs for menopausal women and even non-menopausal women. The results of this study can be used by researchers, especially gerontological nurses, as an efficient model for further theory-based research into Health promotion and improvement.

## Data Availability

The datasets used and analyzed in this study are available from the corresponding author on reasonable request.

## Abbreviations

HLI: Health literacy index
SC: Self-care
QOL: Quality of life
